# Outcomes and toxicity of allogeneic hematopoietic cell transplantation in chronic myeloid leukemia patients previously treated with second-generation tyrosine kinase inhibitors: a prospective non-interventional study from the Chronic Malignancy Working Party of the EBMT

**DOI:** 10.1038/s41409-021-01472-x

**Published:** 2021-10-01

**Authors:** Stavroula Masouridi-Levrat, Eduardo Olavarria, Simona Iacobelli, Mahmoud Aljurf, Elena Morozova, Riitta Niittyvuopio, Henrik Sengeloev, Peter Reményi, Grzegorz Helbig, Paul Browne, Arnold Ganser, Arnon Nagler, John A. Snowden, Marie Robin, Jakob Passweg, Gwendolyn Van Gorkom, Hélène Labussière Wallet, Jennifer Hoek, Henric-Jan Blok, Theo De Witte, Nicolaus Kroeger, Patrick Hayden, Yves Chalandon, Ibrahim Yakoub Agha

**Affiliations:** 1 Hematology Division and Faculty of Medicine, University Hospitals of Geneva, University of Geneva, Geneva, Switzerland; 2 Hammersmith Hospital, London, UK; 3 University of Rome “Tor Vergata”, Rome, Italy; 4 King Faisal Specialist Hospital & Research Centre, Riyadh, Saudi Arabia; 5 First Pavlov State Medical University of St. Petersburg, St. Petersburg, Russia; 6 HUCH Comprehensive Cancer Center, Helsinki, Finland; 7 Bone Marrow Transplant Unit L 4043, Copenhagen, Denmark; 8 Dél-pesti Centrumkórház, Budapest, Hungary; 9 Silesian Medical Academy, Katowice, Poland; 10 Hope Directorate, Dublin, Ireland; 11 Hannover Medical School, Hannover, Germany; 12 Chaim Sheba Medical Center, Tel-Hashomer, Israel; 13 Sheffield Teaching Hospitals NHS Trust, Sheffield, UK; 14 Hopital St. Louis, Paris, France; 15 University Hospital, Basel, Switzerland; 16 Department of Internal Medicine, Division of Hematology, GROW School for Oncology and Developmental Biology, Maastricht University Medical Center, Maastricht, The Netherlands; 17 Hôpital Lyon Sud, Hospices Civils de Lyon, Pierre Bénite, France; 18 EBMT Data Office Leiden, Leiden, The Netherlands; 19 Nijmegen Medical Centre, Radboud University, Nijmegen, Netherlands; 20 Department of Stem Cell Transplantation, University Hospital Hamburg-Eppendorf, Hamburg, Germany; 21 St. James’s Hospital, Dublin, Ireland; 22 CHU de Lille, Univ Lille, INSERM U1286, Infinite, 59000 Lille, France

**Keywords:** Medical research, Chronic myeloid leukaemia

## Abstract

Allogeneic hematopoietic cell transplantation (allo-HCT) remains a treatment option for patients with chronic myeloid leukemia (CML) who fail to respond to tyrosine kinase inhibitors (TKIs). While imatinib seems to have no adverse impact on outcomes after transplant, little is known on the effects of prior use of second-generation TKI (2GTKI). We present the results of a prospective non-interventional study performed by the EBMT on 383 consecutive CML patients previously treated with dasatinib or nilotinib undergoing allo-HCT from 2009 to 2013. The median age was 45 years (18–68). Disease status at transplant was CP1 in 139 patients (38%), AP or >CP1 in 163 (45%), and BC in 59 (16%). The choice of 2GTKI was: 40% dasatinib, 17% nilotinib, and 43% a sequential treatment of dasatinib and nilotinib with or without bosutinib/ponatinib. With a median follow-up of 37 months (1–77), 8% of patients developed either primary or secondary graft failure, 34% acute and 60% chronic GvHD. There were no differences in post-transplant complications between the three different 2GTKI subgroups. Non-relapse mortality was 18% and 24% at 12 months and at 5 years, respectively. Relapse incidence was 36%, overall survival 56% and relapse-free survival 40% at 5 years. No differences in post-transplant outcomes were found between the three different 2GTKI subgroups. This prospective study demonstrates the feasibility of allo-HCT in patients previously treated with 2GTKI with a post-transplant complications rate comparable to that of TKI-naive or imatinib-treated patients.

## Introduction

Tyrosine kinase inhibitors (TKIs) are established as standard-of-care therapy for patients with chronic myeloid leukemia (CML) since the advent of the first TKI, imatinib mesylate, in the late 1990s [1] and the results of IRIS trial that fundamentally revolutionized the management of the disease [2]. Later, the second-generation TKIs (2GTKIs)—particularly dasatinib and nilotinib—have shown extremely encouraging results in the setting of imatinib resistance or intolerance with long-term overall survival (OS) estimated at >70% [3, 4], while in newly diagnosed CML the 5-year cumulative probability for achieving major molecular response is >75%, significantly higher than with imatinib [5, 6]. However, the use of a third-line 2GTKI (switching between dasatinib, nilotinib, and bosutinib) may be of limited benefit with usually not durable responses [7] especially for the patients with primary cytogenetic resistance [8]. Furthermore, the remarkable efficacy of the third-generation TKI, ponatinib, is highly tempered by a significant vascular toxicity [9]. Finally, 15% of the initial CML population will require an alternative treatment [10]. Thus, despite the efficacy of TKIs, allogeneic hematopoietic stem cell transplantation (allo-HCT) remains an effective and potentially curative option [11–13] for patients who fail to respond durably to TKIs or for patients presented with advanced stage disease.

While the use of imatinib seems to have no adverse impact on outcomes after transplant as shown in early studies [14–18], it is still uncertain whether prior use of 2GTKI affects post-transplantation outcomes. Selection of more aggressive or resistant clones could be expected. Furthermore, suboptimal outcomes could result from increased direct drug toxicity [19] or immune dysfunction [20, 21] given the fact that each TKI has multiple off-targets effects. Previous studies [22–26] provided no evidence of detrimental effect of prior allo-HCT 2GTKIs, but they are mainly retrospective analyses with small number of patients. Moreover, they did not address the question of whether dasatinib compared to nilotinib or to the combination of both has a different impact on subsequent allo-HCT. In order to address these issues we conducted a multicenter prospective non-interventional study on behalf of Chronic Malignancy Working Party of the European Group for Blood and Marrow Transplantation (EBMT).

## Patients, materials, and methods

We prospectively registered adult patients with a diagnosis of CML (all phases) who underwent first allo-HCT between December 2009 and September 2013 and had been previously treated with dasatinib or nilotinib regardless of their response to these drugs. A specific data collection form was sent to the transplant centers to capture the relevant information at the appropriate intervals (day +100; 1 year; 2 years after transplant). The data on the prior treatment of patients with 2GTKI were collected retrospectively as part of the medical history. Informed consent to authorize the release of medical information to the EBMT was obtained in all patients in accordance with the principles laid out in the Declaration of Helsinki. A total of 446 patients were registered for participation, but 63 (14.1%) patients were excluded from the study due to unconfirmed eligibility. Finally, 383 patients from 93 EBMT centers in 27 countries were included in the analysis.

### Study endpoints

The primary objective of our study was to evaluate the influence of prior treatment with 2GTKIs in CML patients on engraftment rates and non-relapse mortality (NRM) after allo-HCT. The secondary objectives were to evaluate the effect of 2GTKIs on transplantation-related toxicity—mainly incidence and severity of acute and chronic graft-versus-host disease (GvHD) and hepatic sinusoidal obstruction syndrome (SOS)—overall and disease-free survival and the relapse rate.

### Definitions

Disease status at the start of 2GTKI treatment and at transplant (last disease assessment prior to transplant) was defined according to European LeukemiaNet [11]. More precisely, all patients beyond first chronic phase (>CP1) prior to transplant were patients who had developed blast crisis (BC) beforehand and regained a chronic phase stage after treatment. Patients in accelerated phase (AP) remained in AP regardless of their response to treatment as CP2 can only be achieved after developing BC but not after AP.

The date of engraftment was defined as the first of 3 consecutive days where the absolute neutrophil count was ≥500/μL. Relapse was defined as hematologic relapse in case of previous remission, or progression in case of previous AP or BC. NRM was defined as death without prior relapse or progression. OS was defined as time from transplant to death from any cause, or to last follow-up if alive. Relapse-free survival (RFS) was defined as time from transplant to relapse or progression or to death from any cause. GvHD cases were divided into acute and chronic ones based on the time of onset using a cutoff of 100 days as previously described [27, 28]. EBMT score was calculated according to Gratwohl et al. [29].

### Statistical analysis

Standard non-parametric tests (Mann–Whitney, Kruskall–Wallis, *χ*^2^, or Fisher exact) were used to compare characteristics between groups. Probabilities of OS and RFS were calculated using the Kaplan–Meier method and were compared between the 2GTKIs groups by the log-rank test. Endpoints with competing risks (engraftment, relapse, acute and chronic GvHD, and NRM) were evaluated by cumulative incidence curves and compared between the 2GTKIs groups by Gray’s test. Adjusted analyses were done applying the Cox regression for the (cause-specific) hazards of event. The following variables were initially assessed and considered candidates for the multivariable model if significant at 0.2 level in the univariate analysis: pre-transplantation TKI use, calendar year of transplant, patient’s gender, patient’s age at transplantation, disease status at start of 2GTKI and at transplantation, interval between diagnosis and transplantation, type of donor, donor–recipient gender combination, donor–recipient CMV constellation, graft source, T-cell depletion, intensity of conditioning regimen (RIC vs MAC), TBI given, performance status at transplant, and EBMT risk score. The reported models were checked to be robust with respect to the consideration of a potential center effect and the proportional hazards assumption.

## Results

The 2GTKI used prior allo-HCT was dasatinib for 155 patients (40%), nilotinib for 64 patients (17%), whereas 164 patients (43%) had a sequential treatment of dasatinib and nilotinib with or without bosutinib/ponatinib. Only 5/164 patients of this third group had bosutinib, while 8/164 patients had ponatinib. The majority of patients (306/383, 80%), had imatinib as primary treatment at diagnosis and therefore switched to 2GTKI at a later stage. The median follow-up period after transplantation was 37 months (1–77). Of note, the median follow-up for the dasatinib group was longer (44 months, range 1–77) compared to the nilotinib (36 months, range 3–62) and the combination groups (34 months, range 1–71) showing that dasatinib was the preferred 2GTKI in the earlier years of the study.

### Patient characteristics

The patient characteristics for all patients and the three 2GTKI subgroups are shown in Table 1. The median age was 45 years (18–68). Disease status at the start of 2GTKI treatment was reported for 265 patients: 123 patients (46%) were in first chronic phase (CP1), 67 patients (25%) were in AP or >CP1, and 75 patients (28%) were in BC. Overall disease status at allo-HCT was CP1 in 139 patients (38%), AP or >CP1 in 163 (45%), and BC in 59 (16%). Of note, only 29% of patients who received dasatinib were in CP1 at the start of 2GTKI treatment and at the time of SCT compared with 45% at the start of 2GTKI and 40% at transplant for patients treated with nilotinib. (Supplementary data: cross tables of disease stage at diagnosis vs disease stage at 2GTKI vs disease stage at transplant). The median interval from diagnosis to allo-HCT was 22 months (2–267) and the median interval between starting 2GTKI and allo-HCT (duration of 2GTKI) was 10 months (1–191). The donor was an HLA-identical sibling in 130 cases (35%). Allo-HCT was performed using peripheral blood stem cells (PBSC) in 77% of cases, while 71% of the conditioning regimens were myeloablative. The EBMT score was low (0–2) in 26 (7%), intermediate (3–4) in 216 (62%), and high (5–7) in 107 patients (31%). Of note, the median EBMT score was 4 for each of the three 2GTKI groups. *χ*^2^ test showed that only interval between diagnosis and transplantation and disease status at start of 2GTKI and at transplant had significantly different distribution according to the 2GTKI group.

**Table 1:** Patient, disease, and transplantation characteristics.

	All patients		Dasatinib		Nilotinib		Sequential/other		
Variable	*n* eval	*n* (%)	*n* eval	*n* (%)	*n* eval	*n* (%)	*n* eval	*n* (%)	*P*
*No. of patients*		383		155		64		164	
Age, years, median (range)	383	45 (18–68)	155	45 (18–68)	64	49 (21–67)	164	45 (18–67)	0.46
Male sex	383	251 (65)	155	110 (71)	64	37 (58)	164	104 (63)	0.33
*Karnofsky score at SCT*	357		143		60		154		0.07
>80%		271 (76)		100 (70)		50 (83)		121 (79)	
≤80%		86 (24)		43 (30)		10 (17)		33 (21)	
*Time from diagnosis to allo-HCT*	383		155		64		164		<0.001
≤12 months		93 (24)		55 (35)		15 (23)		23 (14)	
>12 months		290 (76)		100 (65)		69 (77)		141 (86)	
*Disease stage at 2GTKI*	265		108		40		117		<0.001
CP1		123 (46)		31 (29)		18 (46)		74 (63)	
AP or >CP1		67 (26)		31 (29)		11 (27)		25 (21)	
BC		75 (28)		46 (42)		11 (27)		18 (16)	
*Disease stage at allo-HCT*	361		139		62		160		0.07
CP1		139 (39)		41 (30)		25 (40)		73 (46)	
AP or >CP1		163 (45)		73 (52)		26 (42)		64 (40)	
BC		59 (16)		25 (18)		11 (18)		23 (14)	
*Donor*	374		152		62		160		0.39
Identical sibling		130 (35)		59 (40)		20 (32)		51 (32)	
Other		244 (65)		93 (62)		42 (68)		109 (68)	
*Recipient/donor sex match*	380		154		64		162		0.11
Male–female		71 (19)		31 (20)		6 (9)		34 (21)	
Other		309 (81)		123 (80)		58 (91)		128 (79)	
*Recipient/donor CMV status*	374		149		63		162		0.88
neg/neg		95 (25)		37 (25)		16 (25)		42 (26)	
neg/pos		38 (10)		15 (10)		5 (8)		18 (11)	
pos/neg		84 (23)		29 (20)		16 (25)		39 (24)	
pos/pos		157 (42)		68 (45)		26 (42)		63 (39)	
*Stem cell source*	382		155		63		164		0.89
BM		73 (19)		30 (19)		13 (20)		30 (18)	
PBSC		295 (77)		121 (78)		47 (75)		127 (78)	
CB		14 (4)		4 (3)		3 (5)		7 (4)	
*T-cell depletion*	383	219 (57)	155	85 (55)	64	40 (62)	164	94 (57)	0.58
*TBI given*	383	113 (30)	155	56 (36)	64	12 (19)	164	45 (27)	0.28
*Conditioning regimen*	383		155		64		164		0.58
Myeloablative		272 (71)		114 (74)		46 (72)		112 (68)	
Non myeloablative		111 (29)		41 (26)		18 (28)		52 (32)	
*EBMT score*	349		135		60		154		0.68
1		2 (0.5)		2 (1.5)		0 (0)		0 (0)	
2		24 (7)		11 (8)		4 (6.7)		9 (6)	
3		70 (20)		28 (20.7)		13 (21.7)		29 (19)	
4		146 (42)		58 (43)		23 (38.3)		65 (42)	
5		83 (24)		27 (20)		17 (28.3)		39 (25)	
6		22 (6)		8 (6)		2 (3.3)		12 (8)	
7		2 (0.5)		1 (0.7)		1 (1.7)		0 (0)	

EBMT score: disease stage at transplantation (0 for CP1, 1 for accelerated phase or for >CP1, and 2 for blastic crisis), age (0 for <20 years, 1 for 20–40 years, and 2 for >40 years), interval from diagnosis to transplant (0 for <1 year and 1 for ≥1 year), donor type (0 for an HLA-identical sibling and 1 for an unrelated donor), and donor–recipient sex match (1 for female donor for male recipient and 0 for all others).

*CP* chronic phase, *AP* accelerated phase, *BC* blastic crisis, *PBSC* peripheral blood stem cell, *BM* bone marrow, *CB* cordon blood.

#### Primary endpoints

**Fig. 1:**
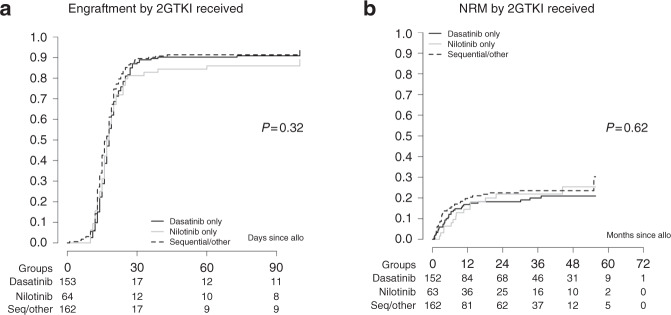
Primary endpoints. No significant differences were observed between the 2GTKI subgroups regarding engraftment (**a**) or non-relapse mortality (NRM) (**b**).

As 2GTKI therapy is associated with adverse hematologic effects, we specifically looked at the impact on engraftment. Engraftment occurrence was evaluated in 379/383 patients. Three hundred and fifty patients (92%) engrafted, while 10 patients (3%) experienced primary graft failure and 19 patients (5%) secondary graft failure. The median time to engraftment was 17 days (range 1–100) with no significant differences between the 2GTKI subgroups, *P* = 0.32 (Fig. 1a). From all the factors listed in the statistical paragraph, only stem cell source enter the Cox model of multivariable analysis with PBSC favoring engraftment (PBSC vs other source HR: 2.35, *P* < 0.001). This analysis revealed no significant differences between the 2GTKI subgroups regarding engraftment occurrence.

Regarding NRM, among 141 deaths at the time of the present analysis, 101 (71.6%) were without prior evidence of relapse. The main causes of NRM included GvHD in 50 patients and infection in 36 patients. The overall NRM was estimated at 18% (95% CI 14–22) at 12 months and 24% (95% CI 19–29) at 5 years. No significant differences between the 2GTKI subgroups were observed in univariate analysis, *P* = 0.62 (Fig. 1b). While several adjustment factors were associated in univariable analysis, only patient gender and performance status entered the Cox model of multivariable analysis. The absence of significant differences between the 2GTKI subgroups was confirmed, whereas only performance status (Karnofsky score <90 vs ≥90) had a statistically significant impact on NRM (HR: 1.67, 95% CI: 1.02–2.73). EBMT risk score (≥5 vs <5) had no significant impact on NRM.

#### Secondary endpoints

##### Transplant-related toxicity

**Fig. 2:**
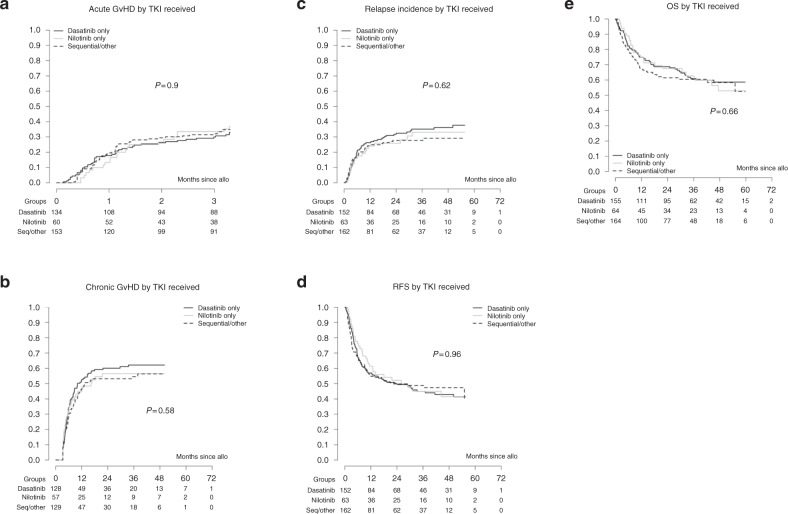
Secondary endpoints. No significant differences were observed between the 2GTKI subgroups regarding acute GvHD (**a**), chronic GvHD (**b**), relapse incidence (**c**), relapse-free survival (RFS) (**d**), and overall survival (OS) (**e**).

Acute GvHD (aGvHD) was evaluable in 347/383 patients (36 patients excluded due to missing data): the incidence of aGvHD grade II–IV was 34% (95% CI 29–39) with a median time to occurrence since transplant of 0.9 months (0.13–3.29 months). No significant difference was observed between the 2GTKI subgroups for aGvHD in univariable analysis (*P* = 0.9) (Fig. 2a). Several adjustment risk factors entered the multivariable model and all of them had a statistically significant impact on the occurrence of aGvHD: donor other than HLA-identical sibling (HR: 2.30, 95% CI: 1.48–3.57), TBI use (HR: 1.73, 95% CI: 1.19–2.53), male patient/female donor vs other combinations (HR: 1.58 95% CI: 1.03–2.44), PBSC as stem cell source (HR: 1.81, 95% CI: 1.09–3.00). However, there were no significant differences between the 2GTKI subgroups and this was confirmed when replacing risk factors by EBMT risk score that itself had a significant impact on aGvHD occurrence (HR: 1.76, 95% CI: 1.21–2.56). Chronic GvHD (cGvHD) was evaluable in 314/383 patients (18 patients excluded due to missing data, 51 because of death, lost to follow-up, or second transplantation before day 100). The 5-year incidence of cGvHD was 60% (95% CI 54–66). cGvHD occurred at a median of 5.7 months (3–61) post transplant. No significant difference between the different 2GTKI subgroups was observed (*P* = 0.58) (Fig. 2b). Hepatic SOS occurred in 6 cases (2%). Even if no comparison between 2GTKI subgroups is possible given the small number, it is noteworthy that 5/6 cases occurred in the dasatinib group, while the 6th case was in the combination group. Out of 299 evaluable patients, 195 (65%) developed a severe infection. There was no difference in infection occurrence between the three different 2GTKI subgroups (*P* = 0.8).

**Fig. 3:**
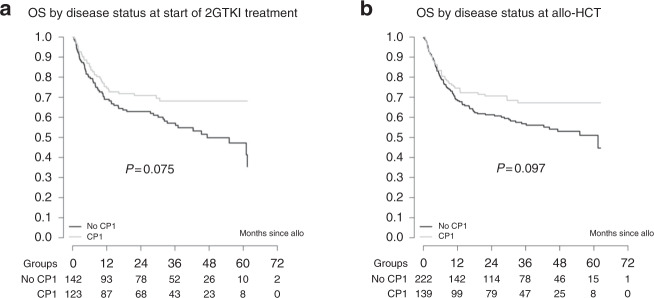
Impact of disease stage on overall survival (OS). Advanced disease stage (defined as other than CP1) at start of 2GTKI treatment (**a**) and at time of allo-HCT (**b**) had a negative impact on OS.

**Fig. 4:**
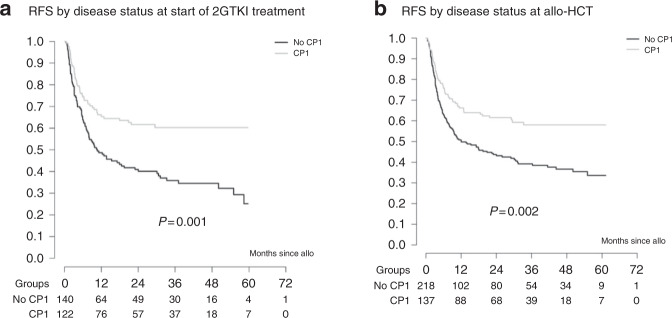
Impact of disease stage on relapse free-survival (RFS). Advanced disease stage (defined as other than CP1) at start of 2GTKI treatment (**a**) and at time of allo-HCT (**b**) had a negative impact on RFS.

**Fig. 5:**
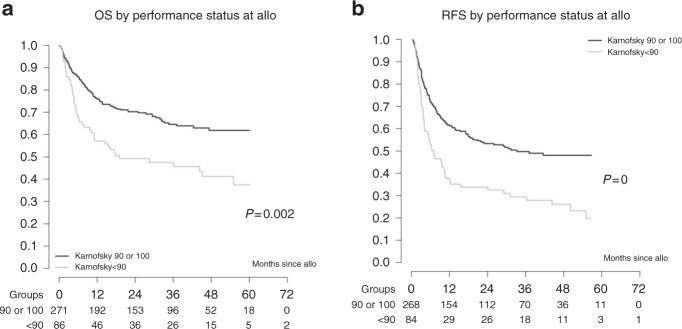
Impact of perfomance status. Poor performance status (defined as Karnofsky < 90) had a negative impact on overall survival (**a**) and relapse-free survival (**b**).

Relapse incidence at 2 and 5 years was estimated at 29% (95% CI 24–33) and 36% (95% CI 29–42), respectively, while RFS was 40% (95% CI 33–47) at 5 years. OS was 65.4% (95% CI 60–70) at 2 years and 56% (95% CI 50–62) at 5 years for all patients. No differences in post-transplant outcomes were found between the three different 2GTKI subgroups (Fig. 2c–e). On univariable analysis, advanced disease stage at start of 2GTKI treatment and at time of allo-HCT had a negative impact on overall (Fig. 3a, b) and RFS (Fig. 4a, b). So, 5-year OS was 67% for patients in CP1 at transplant, 57% for patients in AP or >CP1, and 37% for patients in BC. Poor performance status had also a negative impact on both OS (Fig. 5a) and RFS (Fig. 5b). Multivariable analysis for OS confirmed the absence of difference between 2GTKI groups (nilotinib only vs dasatinib only: HR = 1.16 95% CI: 0.70–1.91 and sequential/other vs dasatinib only: HR = 1.30, 95% CI: 0.88–1.90) in a model adjusted for performance status and disease status at transplant (Table 2, panel A). Similarly, multivariable analysis for RFS confirmed the absence of difference between 2GTKI groups (nilotinib only vs dasatinib only: HR = 0.99 95% CI: 0.58–1.70 and sequential/other vs dasatinib only: HR = 1.28, 95% CI: 0.85–1.92) in a model adjusted for performance status and disease status at transplant and at start of 2GTKI treatment (Table 2, panel B). On multivariable analysis poor performance status maintained its clear negative impact on both OS and PFS. We observed a trend for better OS in patients in CP1 at transplant and a trend for better PFS in patients in CP1 at transplant and those in CP1 at start of 2GTKI. Patients with EBMT risk score <5 showed a non-significant trend toward predicting better outcome (*P* = 0.0628).

**Table 2:** **A** Multivariate analysis of OS according to previous given 2GTKI and **B** multivariate analysis of RFS according to previous given 2GTKI.

	HR	95% CI	*P* value
*A*			
TKI: nilotinib vs dasatinib	1.16	0.70, 1.91	0.567
TKI: seq/other vs dasatinib	1.30	0.88, 1.90	0.186
Status at allo-HCT: CP1 vs no CP1	0.70	0.48, 1.03	0.068
Karnofsky PS: <90 vs 90 or 100	1.90	1.32, 2.75	0.001
*B*			
TKI: nilotinib vs dasatinib	0.99	0.58, 1.70	0.976
TKI: seq/other vs dasatinib	1.28	0.85, 1.92	0.244
Status at allo-HCT: CP1 vs no CP1	0.64	0.41, 1.02	0.062
Status at 2GTKI: CP1 vs no CP1	0.66	0.42, 1.05	0.078
Karnofsky PS: <90 vs 90 or 100	2.23	1.51, 3.29	<0.001

## Discussion

Despite the excellent long-term survival for CML patients diagnosed in CP undergoing TKI treatment and a near normal life expectancy [30], allo-HCT continues to be an important treatment option for some patients [12]. However, the timing of the transplant has changed to third or fourth line after failure of or intolerance to at least one 2GTKI according to current recommendations [11]. Concerns regarding the feasibility and the safety of a subsequent allo-HCT are justified given some well-known side effects of 2GTKI. For instance, myelotoxicity could predispose to engraftment delay or liver toxicity to SOS. Careful evaluation of new therapeutic modalities regarding their potential impact on transplant-related mortality is therefore mandatory.

Our prospective study shows that pre-treatment with 2GTKI does not result in excessive transplant-related toxicity or mortality and does not have a detrimental effect on post-transplantation outcomes. The engraftment rate, the overall and RFS, the incidence of relapse, the NRM, and the rate of post-transplant complications are comparable to those of patients pre-treated with imatinib [13, 16] or TKI-naive [31, 32] patients demonstrating the feasibility of allo-HCT in patients previously treated with 2GTKI. Of note, the observed graft failure rate of 8% is higher than that reported in leukemia patients [33]. Although the primary graft failure rate (3%) was relatively low, it can be hypothesized that CML patients, independently of the type of TKI treatment, present a higher risk of graft failure because of the weak immunosuppressive effect of the disease and its treatment. The relative high rate of graft failure has been previously demonstrated [15, 16] with “only” 88–90% of imatinib-treated patients and 90% of TKI-naive patients achieving engraftment. It should be pointed out, though, that the comparison of outcomes with transplantation of the pre-TKI era is rather speculative because of the evolution of transplant modalities and the achievement of substantial reduction in transplant-related mortality in recent years.

Our results confirm prior observations: during the first 5 years of 2GTKI use, three retrospective studies [22–24] analyzing the outcome of a total of 43 patients who underwent allo-HCT following dasatinib or nilotinib treatment after imatinib failure provided no evidence for increased risk for graft failure or delayed engraftment, treatment-related organ toxicity or GVHD. Comparable results were demonstrated in a later observational prospective analysis of 28 CML patients [25]. Compared to previous studies, a prominent feature of the present analysis is the inclusion of a substantial number of patients treated with 2GTKI in a prospective manner. Moreover, the question about the individual influence of dasatinib and nilotinib has been addressed for the first time in this observational study with the comparison of the outcomes between the three 2GTKI subgroups. As might be expected, combination group patients presented a longer interval between diagnosis and transplantation, suggesting a longer TKI exposure duration. Of note, patients receiving dasatinib were more likely to proceed to allo-HCT in advanced phase than patients receiving nilotinib or both 2GTKI. This can be partially explained by the fact that dasatinib was given more often in earlier years, when ponatinib was not available yet and maintenance of CP before allo-HCT was less feasible [34]. Interestingly, we observed no differences in outcomes between the three 2GTKI subgroups. Our observation is in contrast to that of Kondo et al. [26] who analyzed the data of 237 patients for whom the number of pre-SCT TKI varied from one to three and identified the use of three TKIs before transplantation as a significant and independent adverse risk factor for prognosis because of a higher NRM rate. Although the number of TKIs was not specifically recorded in our study, we can safely assume that the majority of sequential treatment group patients were exposed to at least three TKIs but they did not experience a worse survival. This difference can be partially explained by the fact that in the Japanese study the proportion of patients with advanced disease at transplant was higher in patients with three TKIs than in patients with one TKI, whereas our three 2GTKI subgroups did not differ regarding to disease stage at transplant. It has been shown [16, 35–37] that pre-transplant TKI response impact on the post-transplantation outcome, while according to others [38], poor outcome after allo-HCT is associated with advanced disease at diagnosis but not disease status prior to transplantation. In our study, disease status at transplant was only of borderline significance for OS and RFS. Poor performance status at transplant had significantly negative impact on prognosis, although it is perhaps surprising and difficult to explain that EBMT risk group, based originally on CML data in the pre-TKI era [29], was not significantly predictive of outcome.

In our study, 101/383 patients (26.4%) died from causes other than disease relapse, figure not unexpected. GvHD was the leading cause of death with an incidence comparable to that observed in patients not treated with 2GTKIs [13, 31, 32] (50/383 patients,13%), followed by infections. Given the increasing experience on the efficacy of TKIs as GvHD treatment [39–41], the question of influence of the immunomodulatory potency of TKIs [42–48] on GvHD incidence and severity could be raised. Significantly lower incidence of cGvHD in patients pre-treated with imatinib compared with a historical group control has been reported [15], but it can be attributed to differences in GvHD prophylaxis and mainly a more frequent use of antithymocyte globulin in imatinib group. The lack of a lower GvHD incidence in our study was not surprising, given that the immunomodulatory effects of TKI prior to transplant are not expected to persist post transplantation and even less likely to affect the donor’s origin T-cell function.

Finally, only 2% of patients experienced SOS that is lower than the overall incidence of post-HCT SOS [49, 50] and importantly lower than the incidence of 25% reported previously [25] for pre-treated with 2GTKI patients who proceed to allo-HCT.

There are several caveats to these data, including missing detailed information on molecular or cytogenetic assessment of disease status, mutation status, and the indication for 2GTKI prior to allo-HCT. Information about therapy for relapse post allo-HCT was not analyzed. The absence of data regarding patients’ comorbidities, which could potentially explain the non-significant impact of EBMT score to outcome, is another limitation of our study. Nonetheless, these results highlight the safety and efficacy of allo-HCT after 2GTKIs, with comparable outcomes and no unexpected toxicity.

The time to proceed to allo-HCT remains highly controversial especially for patients still in chronic phase who fail second-line treatment. We consider that our study could be useful in defining risk-adapted strategies for patients with suboptimal response or failure to TKIs. It is obvious that an individualized risk-benefit assessment is needed in each patient, mainly regarding age, comorbidities, molecular aberrations, and donor availability. Despite considerable morbidity and mortality, allo-HCT offers a curative option with a very high survival rate for patients in CP. Given that pre-transplantation 2GTKI treatment does not adversely impact transplantation outcomes and that transplantation results seem better in case of CP1, the strategy to consider transplant before third-line treatment failure and loss of CP1 is a reasonable approach.

## Supplementary information

All members of consortia

Cross tables of disease stage at diagnosis vs disease stage at 2GTKI start vs disease stage at transplant
