# Supplementary material for: Outcomes and toxicity of allogeneic hematopoietic cell transplantation in chronic myeloid leukemia patients previously treated with second-generation tyrosine kinase inhibitors: a prospective non-interventional study from the Chronic Malignancy Working Party of the EBMT
Source: Bone Marrow Transplant. 2021 Oct 1;57(1):23–30. doi: 10.1038/s41409-021-01472-x (PMC8732279; doi:10.1038/s41409-021-01472-x)
Supplement: Supplementary file 1 — All members of consortia [file 41409_2021_1472_MOESM1_ESM.docx]

Eduardo Olavarria, Hammersmith Hospital, London, UK, Mahmoud Aljurf ^,^King Faisal Specialist Hospital & Research Centre, Riyadh, Saudi Arabia, Elena Morozova^,^ First Pavlov State Medical University of St. Petersburg, St. Petersburg, Russia, Riitta Niittyvuopio HUCH Comprehensive Cancer Center, Helsinki, Finland, Henrik Sengeloev Bone Marrow Transplant Unit L 4043 Copenhagen, Denmark, Peter ReményiDél-pesti Centrumkórház – Budapest, Hungary, Grzegorz Helbig^,^ Silesian Medical Academy Katowice Poland, Paul Browne^,^ Hope Directorate Dublin, Ireland, Arnold GanserHannover Medical School, Hannover, Germany, Arnon Nagler Chaim Sheba Medical Center, Tel-Hashomer, Israel, John A. Snowden Sheffield Teaching Hospitals NHS Trust, Sheffield, UK Marie Robin Hopital St. Louis, Paris, France, Jakob Passweg University Hospital, Basel, Switzerland, Gwendolyn Van Gorkom Department of Internal  Medicine, Division of Hematology, GROW School for Oncology and Developmental Biology, Maastricht University Medical Center, Maastricht, The Netherlands, Hélène Labussière Wallet Hôpital Lyon Sud, Hospices Civils de Lyon, Pierre Bénite, France, Theo De Witte Radboud University, Nijmegen Medical Centre, Nijmegen, Netherlands, Nicolaus Kroeger Department of Stem Cell Transplantation University Hospital Hamburg-Eppendorf ,Hamburg, Germany, Patrick Hayden St. James’s Hospital, Dublin, Ireland,

Francesca Bonifazi, Bologna University, S.Orsola-Malpighi Hospital, Bologna, Italy; Kristina Carlson, University Hospital, Uppsala, Sweden; Goda Choi, University Medical Center Groningen (UMCG), Groningen, Netherlands; Jürgen Finke, University of Freiburg, Freiburg, Germany; Polina Stepensky, Hadassah University Hospital, Jerusalem, Israel; Dietrich Beelen, University Hospital, Essen, Germany; Tobias Gedde-Dahl, Oslo University Hospital, Rikshospitalet, Oslo, Norway; Massimo Martino, Grande Ospedale Metropolitano Bianchi Melacrino Morelli - Centro Unico Trapianti A. Neri, Reggio_Calabria, Italy; Christof Scheid, University of Cologne, Cologne, Germany; Herve Tilly, Centre Henri Becquerel, Rouen, France; Manuel Abecasis, Inst. Portugues Oncologia, Lisboa, Portugal; Didier Blaise, Programme de Transplantation&Therapie Cellulaire, Marseille, France; J.L. Byrne, Nottingham University, Nottingham, UK; Charles Craddock, University Hospital Birmingham NHSTrust, Birmingham, UK; Eric Deconinck, Hopital Jean Minjoz, Besancon, France; Roberto Foá, Univ. La Sapienza, Rome, Italy; Achilles Anagnostopoulos, George Papanicolaou General Hospital, Thessaloniki, Greece; Martin Bornhäuser, Universitaetsklinikum Dresden, Dresden, Germany; Peter Dreger, University of Heidelberg, Heidelberg, Germany; Xavier Poiré, Cliniques Universitaires St. Luc, Brussels, Belgium; Mareike Verbeek, Klinikum Rechts der Isar, Munich, Germany; Antonin Vitek, Institute of Hematology and Blood Transfusion, Prague, Czech Rep; Ipek Yonal-Hindilerden, Ýstanbul Tip Fakultesi, Istanbul, Turkey; Tsila Zuckerman, Rambam Medical Center, Haifa, Israel; Jacques-Olivier Bay, CHU ESTAING, Clermont_Ferr, France; Bruno Benedetto, S.S.C.V.D Trapianto di Cellule Staminali, Torino, Italy; Fabio Ciceri, Ospedale San Raffaele s.r.l., Milano, Italy; Cecilia Isaksson, Umea University Hospital, Umea, Sweden; Johan Maertens, University Hospital Gasthuisberg, Leuven, Belgium; Ellen Meijer, VU University Medical Center, Amsterdam, Netherlands; Stephan Mielke, Karolinska University Hospital, Stockholm, Sweden; Ron Ram, Tel Aviv Sourasky Medical Center, Tel_Aviv, Israel; Jorge Sierra, Hospital Santa Creu i Sant Pau, Barcelona, Spain; Matthias Stelljes, University of Muenster, Muenster, Germany; Jörg Cammenga, University Hospital, Linkoeping, Sweden; Mercedes Colorado Araujo, Hospital U. Marqués de Valdecilla, Santander, Spain; Matthias Edinger, University Regensburg, Regensburg, Germany; Edgar Faber, NADACE HAIMOM, Olomouc, Czech Rep; Mathilde Hunault-Berger, CHRU, Angers, France; Ain Kaare, Tartu University Hospital, Tartu, Estonia; Giorgio La Nasa, Centro Trapianti Unico Di CSE Adulti e Pediatrico A. O Brotzu, Cagliari, Italy; Xavier Leleu, Hopital La Miletrie, Poitiers, France; Stig Lenhoff, Skanes University Hospital, Lund, Sweden; Maurizio Musso, Ospedale La Maddalena - Dpt. Oncologico, Palermo, Italy; Josep Maria Ribera Santasusana, ICO-Hospital Universitari Germans Trias i Pujol, Badalona, Spain; Luigi Rigacci, Ospedale S. Camillo-Forlanini, Rome, Italy; Christoph Schmid, Klinikum Augsburg , Augsburg, Germany; Radovan Vrhovac, University Hospital Center Rebro, Zagreb, Croatia; Tomasz Wrobel, Uniwersytecki Szpital Kliniczny, Wroclaw, Poland; Pavel Zák, Charles University Hospital, Hradec_Kralove, Czech Rep; William Arcese, ¨Tor Vergata¨ University of Rome, Rome, Italy; Ali Bazarbachi, Department of Internal Medicine, Beirut, Lebanon; Jose Luis Bello López, Hospital Clinico Universitario , S_de_Compostela, Spain; Yves Bertrand, Institut d`Hematologie et d`Oncologie Pediatrique, Lyon, France; Jean Henri Bourhis, Gustave Roussy Cancer Campus, Villejuif, France; Claude Eric Bulabois, CHU Grenoble Alpes - Université Grenoble Alpes, Grenoble, France; Gandhi Damaj, CHU CAEN, Caen, France; Franca Fagioli, Onco-Ematologia Pediatrica, Torino, Italy; Anne Huynh, CHU - Institut Universitaire du Cancer Toulouse, Toulouse, France; Pavel Jindra, Charles University Hospital, Pilsen, Czech Rep; William Krüger, Klinik fuer Innere Medizin C, Greifswald, Germany; Bruno Lioure, Techniciens d`Etude Clinique suivi de patients greffes, Strasbourg, France; Giuseppe Milone, Ospedale Policlinico, Catania, Italy; Murawski Niels, University of Saarland, Homburg, Germany; Francesco Onida, Fondazione IRCCS - Ca’ Granda, Milano, Italy; Zubeyde Nur Ozkurt, Gazi University Faculty of Medicine, Ankara, Turkey; Alessandro Rambaldi, ASST Papa Giovanni XXIII, Bergamo, Italy; Rik Schots, Universitair Ziekenhuis Brussel, Brussels, Belgium; David Valcárcel, Hospital Vall d`Hebron, Barcelona, Spain.
