# Supplementary material for: Outcomes and toxicity of allogeneic hematopoietic cell transplantation in chronic myeloid leukemia patients previously treated with second-generation tyrosine kinase inhibitors: a prospective non-interventional study from the Chronic Malignancy Working Party of the EBMT
Source: Bone Marrow Transplant. 2021 Oct 1;57(1):23–30. doi: 10.1038/s41409-021-01472-x (PMC8732279; doi:10.1038/s41409-021-01472-x)
Supplement: Supplementary file 2 — Cross tables of disease stage at diagnosis vs disease stage at 2GTKI start vs disease stage at transplant [file 41409_2021_1472_MOESM2_ESM.docx]

**Supplementary data** : By stage at diagnosis, cross tables of stage at 2GTKI start and stage at all–HCT separately per group of 2GTKI and for all the patients of the 3 TKI groups together.

| **Stage at Diagnosis** | **2GTKI group** | | | | **Stage at allo** | | | Total |
| --- | --- | --- | --- | --- | --- | --- | --- | --- |
|  |  |  |  |  | CP1 | AP/>CP1 | BC |  |
| **CP1** | **Dasatinib only** | Stage at 2GTKI | CP1 | n | **18** | **7** | **1** | **26** |
|  |  |  |  | % within stage at 2GTKI | 69.2% | 26.9% | 3.8% | 100.0% |
|  |  |  |  | % within stage at allo | 78.3% | 24.1% | 14.3% | 44.1% |
|  |  |  |  | % of Total | 30.5% | 11.9% | 1.7% | 44.1% |
|  |  |  | AP/ >CP1 | n | **4** | **10** | **2** | **16** |
|  |  |  |  | % within stage at 2GTKI | 25.0% | 62.5% | 12.5% | 100.0% |
|  |  |  |  | % within stage at allo | 17.4% | 34.5% | 28.6% | 27.1% |
|  |  |  |  | % of Total | 6.8% | 16.9% | 3.4% | 27.1% |
|  |  |  | BC | n | **1** | **12** | **4** | **17** |
|  |  |  |  | % within stage at 2GTKI | 5.9% | 70.6% | 23.5% | 100.0% |
|  |  |  |  | % within stage at allo | 4.3% | 41.4% | 57.1% | 28.8% |
|  |  |  |  | % of Total | 1.7% | 20.3% | 6.8% | 28.8% |
|  |  | Total | | n | ***23*** | ***29*** | ***7*** | ***59*** |
|  | **Nilotinib only** | Stage at 2GTKI | CP1 | n | **12** | **2** | **3** | **17** |
|  |  |  |  | % within stage at 2GTKI | 70.6% | 11.8% | 17.6% | 100.0% |
|  |  |  |  | % within stage at allo | 100.0% | 18.2% | 42.9% | 56.7% |
|  |  |  |  | % of Total | 40.0% | 6.7% | 10.0% | 56.7% |
|  |  |  | AP/>CP1 | n | **0** | **4** | **3** | **7** |
|  |  |  |  | % within stage at 2GTKI | 0.0% | 57.1% | 42.9% | 100.0% |
|  |  |  |  | % within stage at allo | 0.0% | 36.4% | 42.9% | 23.3% |
|  |  |  |  | % of Total | 0.0% | 13.3% | 10.0% | 23.3% |
|  |  |  | BC | n | **0** | **5** | **1** | **6** |
|  |  |  |  | % within stage at 2GTKI | 0.0% | 83.3% | 16.7% | 100.0% |
|  |  |  |  | % within stage at allo | 0.0% | 45.5% | 14.3% | 20.0% |
|  |  |  |  | % of Total | 0.0% | 16.7% | 3.3% | 20.0% |
|  |  | Total | | n | ***12*** | ***11*** | ***7*** | ***30*** |
|  | **Combination** | Stage at 2GTKI | CP1 | n | **44** | **18** | **3** | **65** |
|  |  |  |  | % within stage at 2GTKI | 67.7% | 27.7% | 4.6% | 100.0% |
|  |  |  |  | % within stage at allo | 91.7% | 54.5% | 37.5% | 73.0% |
|  |  |  |  | % of Total | 49.4% | 20.2% | 3.4% | 73.0% |
|  |  |  | AP/ >CP1 | n | **4** | **11** | **2** | **17** |
|  |  |  |  | % within stage at 2GTKI | 23.5% | 64.7% | 11.8% | 100.0% |
|  |  |  |  | % within stage at allo | 8.3% | 33.3% | 25.0% | 19.1% |
|  |  |  |  | % of Total | 4.5% | 12.4% | 2.2% | 19.1% |
|  |  |  | BC | n | **0** | **4** | **3** | **7** |
|  |  |  |  | % within stage at 2GTKI | 0.0% | 57.1% | 42.9% | 100.0% |
|  |  |  |  | % within stage at allo | 0.0% | 12.1% | 37.5% | 7.9% |
|  |  |  |  | % of Total | 0.0% | 4.5% | 3.4% | 7.9% |
|  |  | Total |  | n | ***48*** | ***33*** | ***8*** | ***89*** |
|  | **All groups** | Stage at 2GTKI | CP1 | n | **74** | **27** | **7** | **108** |
|  |  |  |  | % within stage at 2GTKI | 68.5% | 25.0% | 6.5% | 100.0% |
|  |  |  |  | % within stage at allo | 89.2% | 37.0% | 31.8% | 60.7% |
|  |  |  |  | % of Total | 41.6% | 15.2% | 3.9% | 60.7% |
|  |  |  | AP/ >CP1 | n | **8** | **25** | **7** | **40** |
|  |  |  |  | % within stage at 2GTKI | 20.0% | 62.5% | 17.5% | 100.0% |
|  |  |  |  | % within stage at allo | 9.6% | 34.2% | 31.8% | 22.5% |
|  |  |  |  | % of Total | 4.5% | 14.0% | 3.9% | 22.5% |
|  |  |  | BC | n | **1** | **21** | **8** | **30** |
|  |  |  |  | % within stage at 2GTKI | 3.3% | 70.0% | 26.7% | 100.0% |
|  |  |  |  | % within stage at allo | 1.2% | 28.8% | 36.4% | 16.9% |
|  |  |  |  | % of Total | 0.6% | 11.8% | 4.5% | 16.9% |
|  |  | Total | | n | ***83*** | ***73*** | ***22*** | ***178*** |
| **AP** | **Dasatinib only** | Stage at 2GTKI | CP1 | n | **1** | **3** | **0** | **4** |
|  |  |  |  | % within stage at 2GTKI | 25.0% | 75.0% | 0.0% | 100.0% |
|  |  |  |  | % within stage at allo | 50.0% | 21.4% | 0.0% | 22.2% |
|  |  |  |  | % of Total | 5.6% | 16.7% | 0.0% | 22.2% |
|  |  |  | AP/ >CP1 | n | **1** | **9** | **1** | **11** |
|  |  |  |  | % within stage at 2GTKI | 9.1% | 81.8% | 9.1% | 100.0% |
|  |  |  |  | % within stage at allo | 50.0% | 64.3% | 50.0% | 61.1% |
|  |  |  |  | % of Total | 5.6% | 50.0% | 5.6% | 61.1% |
|  |  |  | BC | n | **0** | **2** | **1** | **3** |
|  |  |  |  | % within stage at 2GTKI | 0.0% | 66.7% | 33.3% | 100.0% |
|  |  |  |  | % within stage at allo | 0.0% | 14.3% | 50.0% | 16.7% |
|  |  |  |  | % of Total | 0.0% | 11.1% | 5.6% | 16.7% |
|  |  | Total | | n | **2** | **14** | **2** | **18** |
|  | **Nilotinib only** | Stage at 2GTKI | AP/ >CP1 | n | **2** |  |  | **2** |
|  |  |  |  | % within stage at 2GTKI | 100.0% |  |  | 100.0% |
|  |  |  |  | % within stage at allo | 100.0% |  |  | 100.0% |
|  |  |  |  | % of Total | 100.0% |  |  | 100.0% |
|  |  | Total | | n | **2** |  |  | **2** |
|  | **Combination** | Stage at 2GTKI | CP1 | n | **1** | **4** |  | **5** |
|  |  |  |  | % within stage at 2GTKI | 20.0% | 80.0% |  | 100.0% |
|  |  |  |  | % within stage at allo | 100.0% | 30.8% |  | 35.7% |
|  |  |  |  | % of Total | 7.1% | 28.6% |  | 35.7% |
|  |  |  | AP/ >CP1 | n | **0** | **7** |  | **7** |
|  |  |  |  | % within stage at 2GTKI | 0.0% | 100.0% |  | 100.0% |
|  |  |  |  | % within stage at allo | 0.0% | 53.8% |  | 50.0% |
|  |  |  |  | % of Total | 0.0% | 50.0% |  | 50.0% |
|  |  |  | BC | n | **0** | **2** |  | **2** |
|  |  |  |  | % within stage at 2GTKI | 0.0% | 100.0% |  | 100.0% |
|  |  |  |  | % within stage at allo | 0.0% | 15.4% |  | 14.3% |
|  |  |  |  | % of Total | 0.0% | 14.3% |  | 14.3% |
|  |  | Total | | n | **1** | **13** |  | **14** |
|  | **All groups** | Stage at 2GTKI | CP1 | n | **2** | **7** | **0** | **9** |
|  |  |  |  | % within stage at 2GTKI | 22.2% | 77.8% | 0.0% | 100.0% |
|  |  |  |  | % within stage at allo | 40.0% | 25.9% | 0.0% | 26.5% |
|  |  |  |  | % of Total | 5.9% | 20.6% | 0.0% | 26.5% |
|  |  |  | AP/ >CP1 | n | **3** | **16** | **1** | **20** |
|  |  |  |  | % within stage at 2GTKI | 15.0% | 80.0% | 5.0% | 100.0% |
|  |  |  |  | % within stage at allo | 60.0% | 59.3% | 50.0% | 58.8% |
|  |  |  |  | % of Total | 8.8% | 47.1% | 2.9% | 58.8% |
|  |  |  | BC | n | **0** | **4** | **1** | **5** |
|  |  |  |  | % within stage at 2GTKI | 0.0% | 80.0% | 20.0% | 100.0% |
|  |  |  |  | % within stage at allo | 0.0% | 14.8% | 50.0% | 14.7% |
|  |  |  |  | % of Total | 0.0% | 11.8% | 2.9% | 14.7% |
|  |  | Total | | n | ***5*** | ***27*** | ***2*** | ***34*** |
| **BC** | **Dasatinib only** | Stage at 2GTKI | CP1 | n | **0** | **1** | **0** | **1** |
|  |  |  |  | % within stage at 2GTKI | 0.0% | 100.0% | 0.0% | 100.0% |
|  |  |  |  | % within stage at allo | 0.0% | 10.0% | 0.0% | 4.8% |
|  |  |  |  | % of Total | 0.0% | 4.8% | 0.0% | 4.8% |
|  |  |  | AP/>CP1 | n | **1** | **1** | **1** | **3** |
|  |  |  |  | % within stage at 2GTKI | 33.3% | 33.3% | 33.3% | 100.0% |
|  |  |  |  | % within stage at allo | 20.0% | 10.0% | 16.7% | 14.3% |
|  |  |  |  | % of Total | 4.8% | 4.8% | 4.8% | 14.3% |
|  |  |  | BC | n | **4** | **8** | **5** | **17** |
|  |  |  |  | % within stage at 2GTKI | 23.5% | 47.1% | 29.4% | 100.0% |
|  |  |  |  | % within stage at allo | 80.0% | 80.0% | 83.3% | 81.0% |
|  |  |  |  | % of Total | 19.0% | 38.1% | 23.8% | 81.0% |
|  |  | Total | | n | ***5*** | ***10*** | ***6*** | ***21*** |
|  | **Nilotinib only** | Stage at 2GTKI | AP/ >CP1 | n |  | **1** | **1** | **2** |
|  |  |  |  | % within stage at 2GTKI |  | 50.0% | 50.0% | 100.0% |
|  |  |  |  | % within stage at allo |  | 33.3% | 33.3% | 33.3% |
|  |  |  |  | % of Total |  | 16.7% | 16.7% | 33.3% |
|  |  |  | BC | n |  | **2** | **2** | **4** |
|  |  |  |  | % within stage at 2GTKI |  | 50.0% | 50.0% | 100.0% |
|  |  |  |  | % within stage at allo |  | 66.7% | 66.7% | 66.7% |
|  |  |  |  | % of Total |  | 33.3% | 33.3% | 66.7% |
|  |  | Total | | n |  | **3** | **3** | **6** |
|  | **Combination** | Stage at 2GTKI | CP1 | n | **0** | **1** | **1** | **2** |
|  |  |  |  | % within stage at 2GTKI | 0.0% | 50.0% | 50.0% | 100.0% |
|  |  |  |  | % within stage at allo | 0.0% | 50.0% | 14.3% | 20.0% |
|  |  |  |  | % of Total | 0.0% | 10.0% | 10.0% | 20.0% |
|  |  |  | AP/ >CP1 | Count | **0** | **1** | **0** | **1** |
|  |  |  |  | % within stage at 2GTKI | 0.0% | 100.0% | 0.0% | 100.0% |
|  |  |  |  | % within stage at allo | 0.0% | 50.0% | 0.0% | 10.0% |
|  |  |  |  | % of Total | 0.0% | 10.0% | 0.0% | 10.0% |
|  |  |  | BC | n | **1** | **0** | **6** | **7** |
|  |  |  |  | % within stage at 2GTKI | 14.3% | 0.0% | 85.7% | 100.0% |
|  |  |  |  | % within stage at allo | 100.0% | 0.0% | 85.7% | 70.0% |
|  |  |  |  | % of Total | 10.0% | 0.0% | 60.0% | 70.0% |
|  |  | Total | | n | **1** | **2** | **7** | **10** |
|  | **All groups** | Stage at 2GTKI | CP1 | n | **0** | **2** | **1** | **3** |
|  |  |  |  | % within stage at 2GTKI | 0.0% | 66.7% | 33.3% | 100.0% |
|  |  |  |  | % within stage at allo | 0.0% | 13.3% | 6.3% | 8.1% |
|  |  |  |  | % of Total | 0.0% | 5.4% | 2.7% | 8.1% |
|  |  |  | AP/ >CP1 | n | **1** | **3** | **2** | **6** |
|  |  |  |  | % within stage at 2GTKI | 16.7% | 50.0% | 33.3% | 100.0% |
|  |  |  |  | % within stage at allo | 16.7% | 20.0% | 12.5% | 16.2% |
|  |  |  |  | % of Total | 2.7% | 8.1% | 5.4% | 16.2% |
|  |  |  | BC | n | **5** | **10** | **13** | **28** |
|  |  |  |  | % within stage at 2GTKI | 17.9% | 35.7% | 46.4% | 100.0% |
|  |  |  |  | % within stage at allo | 83.3% | 66.7% | 81.3% | 75.7% |
|  |  |  |  | % of Total | 13.5% | 27.0% | 35.1% | 75.7% |
|  |  | **Total** | | **n** | **6** | **15** | **16** | **37** |
